# Modeling of the Accumulated Nitrogen Loss by Volatilization in Topdressing Fertilization in Corn Plants

**DOI:** 10.1002/pei3.70209

**Published:** 2026-09-05

**Authors:** Tales Jesus Fernandes, Eliseu Castanheiro Alberto, Adriele Aparecida Pereira, Adrianne Braga da Fonseca, Douglas Ramos Guelfi Silva

**Affiliations:** ^1^ Department of Statistics Federal University of Lavras Lavras Brazil; ^2^ Statistics and Agricultural Experimentation Maputo Moçambique; ^3^ Soil Science Lavras Brazil; ^4^ Department of Soil Science Federal University of Lavras Lavras Brazil

**Keywords:** ammonia, nitrogen fertilizers, nonlinear regression, sigmoidal pattern

## Abstract

Corn cultivation is one of the most important in the world. Urea possesses a high concentration of nitrogen, which is very important to corn crops, is easily handled, and has a lower cost per kilogram of nitrogen, but it presents high losses of this nutrient through volatilization. Therefore, technologies are developed to minimize these losses, the most widespread being N‐butyl thiophosphoric triamide (NBPT). This study aims to select the most appropriate nonlinear model to describe the accumulated loss of nitrogen by volatilization of urea fertilizers of increased efficiency with NBPT applied as topdressing fertilization in corn plants. Urea treated with NBPT not only reduced, but also retarded the accumulated loss of nitrogen by NH3 volatilization. The von Bertalanffy model best adjusted the data for conventional urea fertilizers, while the Gompertz model best adjusted the data for other fertilizers. The UGRAN fertilizer showed the highest accumulated loss with an estimate of 36.503 kg ha^−1^, registering a loss of approximately 11 kg ha^−1^ of N on the first day after application, while the UNBPT4 fertilizer recorded the lowest accumulated loss with an estimate of 14.623 kg ha^−1^, which corresponds to a reduction of 59.9% in relation to the conventional urea.

## 1 Introduction

Corn cultivation ( *Zea mays* L.) is of great economic and nutritional importance, being widely cultivated in several regions of the world. This cereal plays a crucial role in food production, both for human consumption and animal feed, in addition to being an essential raw material for the biofuel industry (United States Department of Agriculture—USDA 2024). The success of corn farming is directly related to the adoption of appropriate agricultural practices, in which nitrogen fertilization excels as being essential for the growth and productivity of the plant.

Nitrogen (N) is one of the nutrients most required by corn crops, directly influencing their vegetative development and grain production (Santos et al. 2023). However, one of the major difficulties associated with nitrogen fertilization is the efficiency of nitrogen use applied to the soil, considering that a significant part of this nutrient can be lost through volatilization, leaching, or denitrification. Ammonia volatilization is one of the main mechanisms of N loss in Brazilian soils, especially when urea is used as a nitrogen source (Fernandes et al. 2024; Souza et al. 2017; Cassim et al. 2024).

In handling practices, techniques are developed to minimize nitrogen losses and optimize fertilization efficiency, which are extremely important. Topdressing fertilization, for example, emerges as a strategy to improve the efficiency of N absorption, providing the nutrient at critical moments in the plant development cycle. In addition, the use of alternative nitrogen sources, such as stabilized or controlled‐release fertilizers, can be an effective alternative to reduce volatilization losses and increase N availability for the crop, contributing to the development of more efficient and sustainable agricultural practices, aiming to increase productivity and mitigate environmental impacts (Kong et al. 2022; Soares and Cantarella 2023; Santos et al. 2023; Cassim et al. 2024).

In this context, it is extremely important to assess the impacts of topdressing fertilization and different forms of nitrogen application in corn productivity, focused on reducing losses due to ammonia volatilization. The aim is to provide information that contributes to the development of more efficient and sustainable agricultural practices, aiming to increase productivity and mitigate environmental impacts.

The use of high‐efficiency fertilizers, such as urea coated with micronutrients (Zn, B) and urease inhibitors (NBPT), has shown promising results in reducing ammonia (NH_3_) volatilization and increasing agricultural productivity, especially in corn crops (Santos et al. 2021; Adotey et al. 2017). Such physical and chemical coatings stabilize nitrogen (N) release, synchronizing it with plant demand, which minimizes N losses through volatilization (Trenkel 2010). The combination of organic polymers and micronutrients can significantly reduce N losses while improving crop yields.

Minato et al. (2020), da Fonseca et al. (2023), and Santos et al. (2023) used a nonlinear regression model to estimate the loss of N due to the volatilization of nitrogen fertilizers applied to corn. However, these authors used only one model and did not perform a comparison to choose the best model for each type of fertilizer. The N loss pattern has a sigmoidal aspect, so nonlinear models, which include the Logistic, Gompertz, von Bertalanffy, and Brody, are more suitable to describe these biological processes, such as organism growth and accumulated loss, in which the behavior is nonlinear over time (Fernandes et al. 2015; Mischan and Pinho 2014; Silva et al. 2021; Vilas Bôas et al. 2023).

Thus, this article aims to select among the non‐linear Logistic, von Bertalanffy, Gompertz, and Brody models the most appropriate one to describe the accumulated loss of nitrogen by ammonia in different urease inhibitor‐treated urea (NBPT) fertilizers used in topdressing fertilization in corn plants.

## 2 Material and Methods

The study used data from five types of nitrogen fertilizers of increased efficiency with urease inhibitors, such as N‐(n‐butyl) thiophosphoric triamide (NBPT), varying in NBPT concentrations among treatments. The effectiveness of the coatings was evaluated regarding the reduction of ammonia (NH_3_) volatilization. The application of urea coated with NBPT was compared with conventional granulated urea in soils with varied pH. Data from five types of nitrogen fertilizers were extracted from Alberto (2024) and da Fonseca et al. (2023), in an experiment conducted between November 2020 and May 2021, in the municipality of Lavras, Minas Gerais (21°16′00″ S; 44°57′27″ W), in a Red Latosol. The experimental design was done in randomized blocks, with a 5 × 3 + 1 factorial scheme, totaling 48 experimental units.

The optimal NBPT level for coating urea depends on the N fertilizer level applied. Better NBPT effects can be expected in an alkaline pH range, which also favors NH3 volatilization (Ferreira et al. 2022).

The application of nitrogen fertilization in top dressing was done manually at a dose of 150 kg ha^−1^ of nitrogen. The quantification of N‐NH_3_ losses was performed from 13 consecutive collections, with a greater focus on the first weeks after fertilizer application.

The following treatments were used in this study:

- Granulated or conventional urea (UGRAN);
- Urea + NBPT + NPPT (UNBPT1);
- Urea + NBPT fertilizer (UNBPT2);
- Urea + NBPT3 (UNBPT3);
- Urea + NBPT4 (UNBPT4).

Whereas UGRAN is conventional urea and the difference between the other fertilizers is the variation in the concentration of NBPT applied to the urea as shown in Table 1.

**TABLE 1:** Variation in the NBPT concentration in the urea treated for different formulations of urea inhibitors.

Treatments	NBPT concentration (mg kg^−1^)
UGRAN	0
UNBPT1	460
UNBPT2	250
UNBPT3	760
UNBPT4	600

*Source:* da Fonseca et al. (2023).

The equations from four models used to describe the growth curves of the accumulated loss of nitrogen by volatilization in topdressing fertilization in corn plants were: Logistic (1), Gompertz (2), von Bertalanffy (3), and Brody (4).

(1) $$Y_{i}=\frac{\alpha }{1+e^{k\left(\beta -x_{i}\right)}}+\varepsilon_{i}$$

(2) $$Y_{i}=\alpha e^{-e^{k\left(\beta -x_{i}\right)}}+\varepsilon_{i}$$

(3) $$Y_{i}=\alpha {\left(1-\frac{e^{k\left(\beta -x_{i}\right)}}{3}\right)}^{3}+\varepsilon_{i}$$

(4) $$Y_{i}=\alpha \left(1-\beta e^{\left(-kx_{i}\right)}\right)+\varepsilon_{i}$$

where *i* = 1, 2, …, *n*, *n* is the sample size, $Y_{i}$ is the response of the *i*‐th observation of the accumulated loss of N, $x_{i}$ is the independent variable represented by the time in days after application; at this parameterizations, the interpretation of parameters models is: *α* is the expected value (asymptotic) for the accumulated loss of N; *β* is the abscissa of the inflection point, from there on the growth changes from accelerated to decelerated, except for the Brody model that does not have an inflection point; *k* is the maturity index, associated with growth, the higher its value, the less time it takes for the accumulated loss of N to reach the asymptotic value (*α*); $\varepsilon_{i}$ is the random error associated with the i‐th observation that is assumed as $\varepsilon_{i}\sim N\left(0,\;\sigma^{2}\right)$.

Parameter were estimates by the least squares method, utilizing the Gauss‐Newton convergence algorithm. The initial values for the parameter vector $\theta =\left(\alpha^{0},\;\beta^{0},k^{0}\right)$ were obtained through visual analysis of the data in a scatter diagram using the R software (R Core Team 2026) and made available in Supplementary Code S1.

Residual analysis tests, such as Shapiro–Wilk, Breusch‐Pagan, and Durbin‐Watson, were used to evaluate the assumptions about the residuals of the adjusted models. After checking the assumptions, the confidence intervals for the parameters were constructed, which are given by:

$$\mathrm{CI}\;{\left(\theta_{i}\right)}_{1-\alpha }=\hat{\theta_{i}}\pm t_{\left(v,\frac{\alpha }{2}\right)}\sqrt{\hat{\mathrm{Var}}\left(\hat{\theta_{i}}\right)}$$

where $\theta_{i}$ corresponds to the parameter estimate $\theta_{i}$; *v* is the number of freedom degrees $\left(v=n-p\right)$ of the residue; $t_{\left(v,\frac{\alpha }{2}\right)}$ is the upper quantile of the t distribution from Student; *α* is the significance level; $\hat{\mathrm{Var}}\left(\hat{\theta_{i}}\right)$ is the variance estimate of the parameter estimate $\theta_{i}$ obtained from the diagonal of the asymptotic matrix of variances and covariances and CI are the results found for the inferior (IL) and superior (SL) limits (Draper and Smith 1998).

The comparison and selection of the model that best described the data was made based on the adjusted coefficient of determination ($R_{\mathrm{aj}}^{2}$), Akaike information criterion (AIC) and residual standard deviation (RSD).

The estimates of the model parameters, the statistical tests, the figures, the residual analysis, and the verification of the quality of adjustment for model selection in this paper were made using the statistical software R (R Core Team 2026).

## 3 Results and Discussion

The parameters of the nonlinear Logistic, Gompertz, von Bertalanffy, and Brody models were estimated to describe the cumulative loss of nitrogen by volatilization in corn plants using different nitrogen fertilizers. The verification of the assumptions of the residuals revealed that normality and homoscedasticity were met for all the models and treatments. However, some models presented problems with the independence of the residuals. For these cases, as suggested by Silva et al. (2021), an autoregressive structure (parameters $\phi_{1}$ and $\phi_{2}$) was incorporated to model this residual dependence and the metrics of the adjustment quality were recalculated, presented in Table 2.

**TABLE 2:** Evaluators of the adjustment quality, adjusted coefficient of determination ($R_{\mathrm{aj}}^{2}$), Akaike information criterion (AIC) and residual standard deviation (RSD) with autoregressive error structure of the Logistic, Gompertz, von Bertalanffy, and Brody models.

Treatment	Model	Evaluators		RSD
		$R_{\mathrm{aj}}^{2}$	AIC	
UGRAN	Logistic	0.9243	17.3391	2.1479
	Gompertz	0.9917	19.9930	0.7688
	**von Bertalanffy**	**0.9935**	**14.8955**	**0.6899**
	Brody	0.9935	32.0061	0.6946
UNBPT1	Logistic	0.9919	36.2064	0.8164
	**Gompertz**	**0.9962**	**27.5472**	**0.5851**
	von Bertalanffy	0.9828	46.5423	1.2150
	Brody	0.9014	53.8030	3.1140
UNBPT2	Logistic	0.9830	38.76797	0.9009
	Gompertz	0.9934	26.9795	0.5600
	**von Bertalanffy**	**0.9951**	**22.4824**	**0.4904**
	Brody	0.9565	52.5093	1.5280
UNBPT3	Logistic	0.9947	30.9154	0.6660
	**Gompertz**	**0.9975**	**22.0894**	**0.4743**
	von Bertalanffy	0.9914	38.1422	0.8795
	Brody	0.9180	67.0456	2.6730
UNBPT4	Logistic	0.9973	9.2586	0.2896
	**Gompertz**	**0.9985**	**2.9139**	**0.2269**
	von Bertalanffy	0.9965	14.0672	0.3484
	Brody	0.9479	48.5651	1.3130

*Note:* Bold values indicate the best models, as discussed in the text.

*Source:* Authors (2026).

The von Bertalanffy model performed best in describing the data for the UGRAN and UNBPT2 treatments, with lower AIC and RSD values and higher adjusted *R* ^2^. The Gompertz model excelled for the UNBPT1, UNBPT3, and UNBPT4 treatments. Therefore, these two models are the most suitable for describing the loss of N by volatilization in the respective fertilizers. This division among the most suitable models makes sense, since the UGRAN is conventional Urea and the UNBPT2 is the fertilizer with the lowest concentration of NBPT, according to Table 1. In accordance with the definition of the von Bertalanffy model, it theoretically presents the inflection point before the Gompertz model. Fertilizers with a higher concentration of NBPT have a later inflection point compared to those with less NBPT, and naturally the Gompertz model fits better, since NBPT is used precisely to retard nitrogen loss through ammonia volatilization as stated by authors such as Souza et al. (2017), da Fonseca et al. (2023), Santos et al. (2023), Cassim et al. (2024) among others.

Table 3 presents the estimates of the parameters from the models that best fit the data from the five treatments under study: UGRAN, UNBPT1, UNBPT2, UNBPT3, and UNBPT4 tested by Student's *t*‐test, and all parameters were significant at the 1% significance level.

**TABLE 3:** Estimates for the parameters with autoregressive error structure, when necessary, and the respective confidence intervals for parameters of the von Bertalanffy and Gompertz models in the respective fertilizers.

Model	Treatment	Parameter	CI inf. lim.	Estimate	CI sup. lim.
von Bertalanffy	UGRAN	*α*	35.596	36.503	37.411
		*β*	1.151	1.228	1.306
		*k*	0.994	1.078	1.162
		$\phi_{1}$		0.725	
	UNBPT2	*α*	18.717	18.967	19.216
		*β*	3.751	3.830	3.909
		*k*	0.374	0.399	0.425
		$\phi_{1}$		−0.417	
		$\phi_{2}$		−0.525	
Gompertz	UNBPT1	*α*	21.225	21.793	22.372
		*β*	4.447	4.596	4.742
		*k*	0.661	0.782	0.935
	UNBPT3	*α*	21.900	22.391	22.893
		*β*	4.720	4.858	4.995
		*k*	0.529	0.597	0.678
	UNBPT4	*α*	14.372	14.623	14.879
		*β*	4.531	4.650	4.768
		*k*	0.427	0.462	0.502

*Source:* Authors (2026).

According to Table 3, based on the values of the parameter *α* estimates, we can generally state that urea fertilizers treated with NBPT reduced the accumulated losses of nitrogen by volatilization compared to untreated urea. According to Guelfi (2017), urea treated with NBPT can promote up to 79% reduction in losses by volatilization in corn cultivation areas compared to conventional urea.

Table 3 shows that the *β* estimates for fertilizers with NBPT (around the fourth and fifth day) are higher compared to the conventional urea (approximately on the first day). Thus, urea treated with NBPT retarded the accumulated loss of nitrogen by volatilization compared to the conventional urea. The NBPT fulfilled its purpose as mentioned by several authors such as Santos et al. (2023) and Cassim et al. (2024), which is to promote reduction and retard loss, thus leaving the nutrient available in the soil for longer to be incorporated by the plant roots. These model estimates directly corroborate the results of Souza et al. (2017), who state that the urea treated with NBPT, in addition to reducing, also promotes a retard in N losses by volatilization in topdressing nitrogen fertilization. According to the authors, approximately 50% of the total N lost through volatilization occurs in less than 4 days in soils with a pH of 4.5, but in 8–11 days in soils with a pH above 5.4.

According to Soares and Cantarella (2023), the peak of NH3 loss in the treatment with conventional urea is higher and occurs earlier than with Urea + NBPT, which corroborates the higher values for the estimates of the parameters *α* and *β* found in this paper.

Conventional Urea (UGRAN) presented a higher value of accumulated nitrogen loss by volatilization (*α* = 36.503 kg ha^−1^) and with the maximum loss rate recorded approximately on the first day (*β* = 1.228 day), and compared to the urea treated with UNBPT4, it reduced by more than half the accumulated nitrogen loss by volatilization (*α* = 14.623 kg ha^−1^) with the maximum loss rate recorded approximately on the fifth day (*β* = 4.650 day). Therefore, in addition to losing less, the fertilizer UNBPT4 retarded the accumulated loss of nitrogen by volatilization. Based on the estimate of the maximum loss for conventional urea regarding the applied N dose, it is possible to state that there was a loss of approximately 25% (36.5/150) regarding the applied dose. Corroborating the results of Silva et al. (2017), they claim that the average N losses due to NH_3_ volatilization in Urea vary from 15% to 35% of the total N applied.

However, according to Cantarella et al. (2018), depending on climate conditions, soil properties, and agricultural practices, N losses can be even greater and reach 40%–60% of N. Authors such as Trenkel (2010) and Silva et al. (2017) state that NBPT reduces NH_3_ losses by volatilization approximately 60% and can subsequently increase crop yield. Guelfi (2017) indicates that research results in corn cultivation areas showed that N loss by volatilization of urea + NBPT was approximately 8% of the N applied. Thus, for a dose of 150 kg ha^−1^ of N applied as top dressing, approximately 12 kg ha^−1^ would be lost by volatilization, corroborating our results in which 14 kg ha^−1^ of N were lost in 150 kg ha^−1^ applied from the UNBPT4 treatment.

The confidence intervals for *α*, *β*, and *k* parameters in the von Bertalanffy model do not overlap, indicating that there is at least 95% confidence that the true parameters estimate from the UGRAN and UNBPT2 treatments are not equal. Furthermore, the confidence intervals of the UNBPT1 and UNBPT3 treatments do not overlap with the confidence intervals of the parameters *α*, *β*, and *k* estimates of the NBPT4 treatment, indicating that there is a significant difference between the estimates of the UNBPT1 and UNBPT3 parameters compared to the estimates of the UNBPT4 treatment parameters. As stated by Salvador et al. (2025), this is a statistical methodology commonly used to compare treatments based on the comparison of the confidence intervals for the estimate of the parameters from the model.

According to da Fonseca et al. (2023), the greater efficiency of the UNBPT4 treatment compared to other urease inhibitor technologies may be related to the solvent used in the preparation of NBPT. This solvent probably provided greater protection of the NBPT molecule against degradation by factors such as soil temperature and pH during the evaluation days (Engel et al. 2015; Soares and Cantarella 2023).

It is worth remembering that, as mentioned by Vilas Bôas et al. (2023), the abscissa of the inflection point represents the day on which the maximum loss occurs and the ordinate of the inflection point indicates the amount of N lost up to that day. Thus, we have:

- UGRAN, von Bertalanffy Model:
$$\hat{Y}=\hat{\alpha }{\left(\frac{2}{3}\right)}^{3}=36,503{\left(\frac{2}{3}\right)}^{3}=10,816\;\mathrm{kg}\;{\mathrm{ha}}^{-1}.$$

This is the accumulated loss approximately on the first day ($\hat{\beta }=1.228$ days), in which the maximum loss rate occurred for the UGRAN fertilizer, that is, the granulated urea fertilizer had already lost approximately 11 kg of N per hectare approximately 1 day after application. Thus, it is the fertilizer with the greatest loss until the inflection, even though this occurred on the first day;

b UNBPT1, Gompertz Model:
$$\hat{Y}=\frac{\hat{\alpha }}{e}=\frac{21,793\;}{e}=8017\;\mathrm{kg}\;{\mathrm{ha}}^{-1}.$$

This is the accumulated loss approximately up to the fifth day ($\hat{\beta }=4.596$ days), in which the maximum loss rate occurred for the UNBPT1 fertilizer, that is, the UNBPT1 fertilizer had already lost approximately 8 kg of N per hectare approximately 5 days after application;

c UNBPT2, von Bertalanffy Model:
$$\hat{Y}=\hat{\alpha }{\left(\frac{2}{3}\right)}^{3}=18,967{\left(\frac{2}{3}\right)}^{3}=5620\;\mathrm{kg}\;{\mathrm{ha}}^{-1}.$$

This is the accumulated loss approximately up to the fourth day ($\hat{\beta }=3.830$ days) in which the maximum loss rate occurred for the UNBPT2 fertilizer, that is, the UNBPT2 fertilizer had already lost approximately 6 kg of N per hectare approximately 4 days after application;

d UNBPT3, Gompertz Model:
$$\hat{Y}=\frac{\hat{\alpha }}{e}=\frac{22,391\;}{e}=8237\;\mathrm{kg}\;{\mathrm{ha}}^{-1}.$$

This is the accumulated loss approximately up to the fifth day ($\hat{\beta }=4.858$ days) in which the maximum loss rate occurred for the UNBPT3 fertilizer, that is, the UNBPT3 fertilizer lost approximately 8 kg of N per hectare approximately 5 days after application;

e UNBPT4, Gompertz Model:
$$\hat{Y}=\frac{\hat{\alpha }}{e}=\frac{14,623\;}{e}=5380\;\mathrm{kg}\;{\mathrm{ha}}^{-1}.$$

This is the accumulated loss approximately up to the fifth day ($\hat{\beta }=4.650$ days) in which the maximum loss rate occurred for the UNBPT4 fertilizer, that is, the UNBPT4 fertilizer lost approximately 5 kg of N per hectare approximately 5 days after application. Therefore, it is the fertilizer with the lowest accumulated loss up to the inflection point.

**FIGURE 1:**
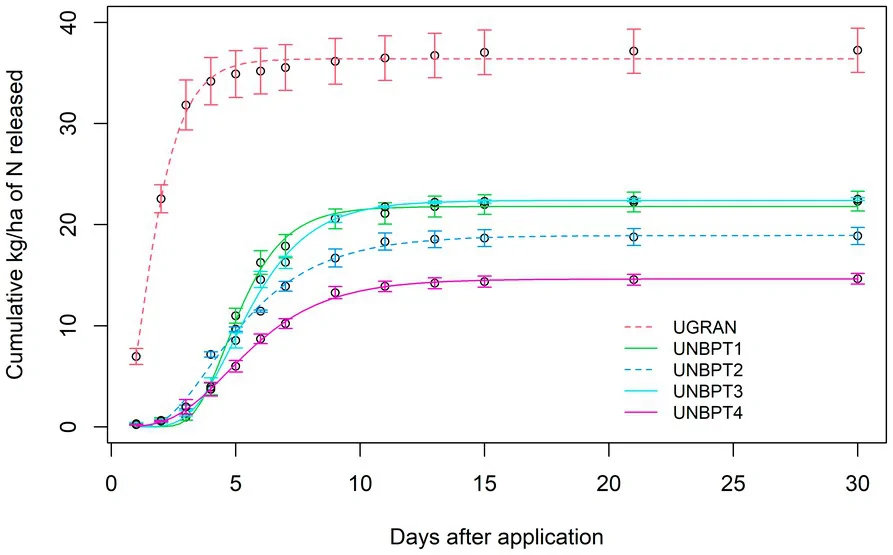
Adjustment of the von Bertalanffy (dashed line) and Gompertz (solid line) models for the accumulated loss of N by volatilization of N‐NH3 in different fertilizers. *Source:* Authors (2026).

Figure 1 presents the graphics of adjustment of the von Bertalanffy model that best described the data from the UGRAN and UNBPT2 treatments and the Gompertz model that best described the data from the UNBPT1, UNBPT3, and UNBPT4 treatments. A visual analysis of the graphics indicates that the models adjusted well to the data. UGRAN and UNBPT2 have a more pronounced loss immediately after application, whereas for the others this loss occurs a little later.

The results corroborate previous studies indicating that urea treated with NBPT reduces and retards nitrogen losses by volatilization. The difference in the parameters of accumulated loss (*α*) and the time until the loss peak (*β*) among treatments confirms the effectiveness of urease inhibitors in improving nitrogen use efficiency. The application of the mixture of increased efficiency urea and conventional urea at adequate N rates not only results in high grain yields but also in increased ecological economic benefits while mitigating environmental impacts (Ma et al. 2022).

Considering the estimate of the parameter *α* (Table 3), the relative efficiency of N loss from other fertilizers in relation to conventional urea was calculated:

- UNBPT1 $\left(36,503-21,793\right)/36,503=0.403=40.3\%$;
- UNBPT2 $\left(36,503-18,967\right)/36,503=0.480=48.0\%$;
- UNBPT3 $\left(36,503-22,391\right)/36,503=0.387=38.7\%$;
- UNBPT4 $\left(36,503-14,623\right)/36,503=0.599=59.9\%$.

The savings in nitrogen loss compared to conventional Urea were 40.3% for UNBPT1, 48.0% for UNBPT2, 38.7% for UNBPT3, and 59.9% for UNBPT4. Note that the most efficient treatment in reducing losses compared to conventional Urea was UNBPT4, reducing 59.9%, as also observed by authors such as Trenkel (2010) and Silva et al. (2017). In their studies, Kong et al. (2022) showed that urea with humic acid reduced N losses by 23.07% due to NH_3_ volatilization compared to the conventional Urea.

The adjusted models provided a detailed view of the dynamics of nitrogen loss through volatilization, evidencing the superiority of fertilizers treated with NBPT over conventional urea. These results have significant practical implications for the management of nitrogen fertilization, reinforcing that the use of urease inhibitors results in nitrogen savings and improves fertilization efficiency. They can also help producers identify the appropriate time to promote fertilization and, according to Sarkis et al. (2021), meet the premises of sustainable production through the 4R concept (right source, right dose, right place, and right time).

## 4 Conclusions

Urea treated with NBPT demonstrated excellent capacity to reduce and retard the accumulated loss of nitrogen by volatilization, evidenced by the estimates of the parameters *α* and *β* from the nonlinear models compared to the estimates for conventional urea.

The von Bertalanffy model was the one that best described the accumulated losses of nitrogen by ammonia volatilization for the UGRAN and UNBPT2 treatments, while the Gompertz model was the most suitable for the fertilizers UNBPT1, UNBPT3, and UNBPT4.

The parameter *α* show that conventional urea (UGRAN) presented the greatest accumulated loss of nitrogen, losing about 11 kg h^−1^ on the first day after application. While the fertilizer UNBPT4 presented the smallest accumulated loss, with a reduction of 59.9% in relation to the conventional urea.

## Funding

This work was supported by Coordination for the Improvement of Higher Education Personnel (CAPES).

## Conflicts of Interest

The authors declare no conflicts of interest.

## Supporting information


**Data S1:** Supplementary code.

## Data Availability

The data and codes that support the findings of this study are available in the Supporting Information of this article.
