# Supplementary material for: Modeling of the Accumulated Nitrogen Loss by Volatilization in Topdressing Fertilization in Corn Plants
Source: Plant Environ Interact. 2026 Sep 5;7(5):e70209. doi: 10.1002/pei3.70209 (PMC13545641; doi:10.1002/pei3.70209)
Supplement: Supplementary file 1 [file PEI3-7-e70209-s001.pdf]

The data is available here.

The R code is presented below.

```
#################################################################

 rm(list=ls())
 library(car)
 library(lmtest)
 library(qpcR)
 library(nlme)

dados <-read.table("Lavras.txt",h=T)
names(dados)
###### UGRAN
plot(dados$DAA,dados$Ureia, pch=1, col=2)
gomp1<-nls(Ureia~A*exp(-(exp(k*(B-DAA)))),start=c(A=40,B=0.5,k=0.09), data=dados)
summary(gomp1)
###  residual analysis   ###
# NORMALIDADE
shapiro.test(residuals(gomp1))
# INDEPENDÊNCIA
MG1=attr(gomp1$m$fitted(),"gradient")
mlg1=lm(Ureia~-1+MG1, data=dados)
durbinWatsonTest(mlg1)
# HOMOCEDASTICIDADE
bptest(mlg1)
pacf(resid(gomp1))
gomp1 <- gnls(Ureia~A*exp(-(exp(k*(B-DAA)))), start=c(A=40,B=0.5,k=0.09),data=dados,
correlation = corARMA(p=1,q=0))
summary(gomp1)
Rsq.ad(gomp1)
AIC(gomp1)
plot(Ureia~DAA, data=dados,
    xlab="Days after application",
    ylab="Cumulative kg/ha of N released")
A <- 36.32960 ; B <-  1.40154;k <- 1.21191
curve(A*exp(-(exp(k*(B-x)))), add=TRUE, col=3, lty=2)
ber1 <- nls(Ureia~a*(1-exp(k*(b-DAA))/3)^3, start=c(a=20,k=0.8,b=0.5),data=dados)
summary(ber1)
###  residual analysis   ###
# NORMALIDADE
shapiro.test(residuals(ber1))
# INDEPENDÊNCIA
MG1=attr(ber1$m$fitted(),"gradient")
mlg1=lm(Ureia~-1+MG1, data=dados)
durbinWatsonTest(mlg1)
# HOMOCEDASTICIDADE
bptest(mlg1)
```

```
pacf(resid(ber1))
ber1 <- gnls(Ureia~a*(1-exp(k*(b-DAA))/3)^3, start=c(a=20,k=0.8,b=0.5),data=dados,
correlation = corARMA(p=1,q=0))
summary(ber1)
Rsq.ad(ber1)
AIC(ber1)
confint(ber1)
plot(Ureia~DAA, data=dados,
    xlab="Days after application",
    ylab="Cumulative kg/ha of N released",
    main="von Bertalanffy:UGRAN")
a <-36.40821; b <- 1.22360; k <-  1.06159
curve(a*(1-exp(k*(b-x))/3)^3, add=TRUE, col=2)
log1=nls(Ureia ~ A/(1+exp(k*(B-DAA))),start=c(A=20,B=2,k=0.8), data=dados)
summary(log1)
###  residual analysis   ###
# NORMALIDADE
shapiro.test(residuals(log1))
# INDEPENDÊNCIA
MG1=attr(log1$m$fitted(),"gradient")
mlg1=lm(Ureia~-1+MG1, data=dados)
durbinWatsonTest(mlg1)
# HOMOCEDASTICIDADE
bptest(mlg1)
pacf(resid(log1))
log1=gnls(Ureia ~ A/(1+exp(k*(B-DAA))),start=c(A=20,B=2,k=0.8), data=dados,  correlation =
corARMA(p=1,q=0))
summary(log1)
Rsq.ad(log1)
AIC(log1)
plot(Ureia~DAA, data=dados,
    xlab="Days after application",
    ylab="Cumulative kg/ha of N released")
A <- 36.14672; B <-  1.76075;  k <- 1.73204
curve(A/(1+exp(k*(B-x))), add=TRUE, col=1)
brod <- nls(Ureia~a*(1-b*exp(-k*DAA)),start=c(a=40,b=0.5,k=0.02477), data=dados)
summary(brod)
###  residual analysis   ###
# NORMALIDADE
shapiro.test(residuals(brod))
# INDEPENDÊNCIA
MG1=attr(brod$m$fitted(),"gradient")
mlg1=lm(Ureia~-1+MG1, data=dados)
durbinWatsonTest(mlg1)
# HOMOCEDASTICIDADE
bptest(mlg1)
plot(Ureia~DAA, data=dados,
    xlab="Days after application",
```

```
    ylab="Cumulative kg/ha of N released")
ab<-36.58690; bb <-1.82219;  kb <- 0.80690
curve(ab*(1-bb*exp(-kb*x)), add=TRUE, col=4)
# comparacao (juntos)
plot(Ureia~DAA, data=dados,
    xlab="Days after application",
    ylab="Cumulative kg/ha of N released")
# gompertz
A <- 36.32960 ; B <-  1.40154;k <- 1.21191
curve(A*exp(-(exp(k*(B-x)))), add=TRUE, col=3, lty=2)
#bertalanffy
a <-36.40821; b <- 1.22360; k <-  1.06159
curve(a*(1-exp(k*(b-x))/3)^3, add=TRUE, col=2)
#logistico
Ag <- 36.14672; Bg <-  1.76075;  kg <- 1.73204
curve(Ag/(1+exp(kg*(Bg-x))), add=TRUE, col=1)
#brody
ab<-36.58690; bb <-1.82219;  kb <- 0.80690
curve(ab*(1-bb*exp(-kb*x)), add=TRUE, col=4)
###  Qualidade de ajuste  ###
Rsq.ad(brod)
AIC(brod)

### And the same applies to the other fertilizers UNBPT1, UNBPT2, UNBPT3, UNBPT4###
```
